# Hydrocarbon Exposure in Myocarditis: Rare Toxic Cause or Trigger? Insights from a Biopsy-Proven Fulminant Viral Case and a Systematic Literature Review

**DOI:** 10.3390/ijms26094006

**Published:** 2025-04-24

**Authors:** Andrea S. Giordani, Tommaso Simone, Anna Baritussio, Cristina Vicenzetto, Federico Scognamiglio, Filippo Donato, Luca Licchelli, Luisa Cacciavillani, Chiara Fraccaro, Giuseppe Tarantini, Fausto Braccioni, Stefania Rizzo, Monica De Gaspari, Cristina Basso, Renzo Marcolongo, Alida L. P. Caforio

**Affiliations:** 1 Cardiology, Department of Cardiac, Vascular, Thoracic Sciences and Public Health, University of Padua—Via Giustiniani 2, 35128 Padua, Italyalida.caforio@unipd.it (A.L.P.C.); 2 Respiratory Pathophysiology Division, Department of Cardiac, Thoracic, Vascular Sciences and Public Health, University of Padova, 35131 Padova, Italy; 3 Cardiovascular Pathology, Department of Cardiac, Thoracic, Vascular Sciences and Public Health, University of Padova, 35131 Padova, Italy

**Keywords:** toxic myocarditis, hydrocarbons, endomyocardial biopsy, fulminant myocarditis, myocarditis prognosis

## Abstract

Toxic myocarditis (TM) is rare, and no systematic evidence is available regarding its treatment or prognosis. Hydrocarbons even more rarely cause TM, and they are associated with severe extracardiac toxicity. Moreover, a pathogenic interaction between viral and toxic agents in TM has not been studied. We present the first case of biopsy-proven parvovirus B19 (B19V) viral fulminant myocarditis diagnosed after hydrocarbon exposure, along with a systematic literature review of hydrocarbon-TM cases. A systematic literature review was conducted by searching hydrocarbon-TM cases. Clinical and prognostic data were recorded. After screening of 937 records, 7 were included. All cases were male, with a median age of 24 years (IQR 23–25). Chest pain and dyspnea were the main symptoms, but arrhythmic presentation was also reported; endomyocardial biopsy (EMB) was performed in only one case. Overall, treatment was based on supportive measures, such as antiarrhythmic and/or vasoactive therapy. Our example (male, 47 years old) is the first reported fulminant biopsy-proven case diagnosed after a massive exposure to hydrocarbons, in which EMB molecular analysis unexpectedly revealed B19V with a high viral load. Hemodynamic and arrhythmic instability required percutaneous stellate ganglion blockade and temporary wearable defibrillator use. Left ventricular function spontaneously normalized at 3 months. In conclusion, we report the first fulminant B19V myocarditis case temporally associated with aromatic hydrocarbon exposure due to a coexistence of viral and toxic causes. Our case and the systematic review show that promptly performing EMB can provide a definitive diagnosis and guide treatment, especially in severe cases in which infectious agents may contribute to myocardial damage.

## 1. Introduction

Myocarditis is an inflammatory disease of the myocardium, defined by histological, immunological, and immunohistochemical criteria. Definitive and etiological diagnosis is based upon endomyocardial biopsy (EMB) examination, which should include polymerase chain reaction (PCR) evaluation for viral genome detection [1]. In the absence of histological data, a presumptive diagnosis of clinically suspected myocarditis is possible, based on the exclusion of alternative causes, such as coronary artery disease, and the evaluation of clinical and imaging findings, essentially non-invasive myocardial characterization by cardiac magnetic resonance (CMR). Myocarditis may exhibit both a heterogeneous etiology and a clinical course; in fact, it may resolve spontaneously, as occurs for most infarct-like myocarditis cases with preserved baseline left ventricular ejection fraction (LVEF), or slowly progress towards an irreversible dilated cardiomyopathy phenotype with LV dilatation and dysfunction, or even present as fulminant myocarditis, i.e., requiring emergent hemodynamic and/or antiarrhythmic support due to cardiogenic shock and/or arrhythmic storm.

The main causes of myocarditis in Western countries are infectious agents, essentially viruses, rather than bacteria, as well as autoimmune/immune mechanisms, as is the case for virus-negative myocarditis [2]. Since non-invasive diagnostic modalities cannot rule out the presence of infectious agents in the myocardium, EMB is remains the sole method for achieving myocarditis etiological diagnosis. Performing EMB allows for the detection, quantification, and characterization of the inflammatory cellular infiltrate, displaying a strong prognostic value *per se* [3], as well as allowing for the precise characterization and quantification of the presence and activity of viral agents and the evaluation of inflammatory cellular markers [4,5,6]. Moreover, when a specific treatment for myocarditis is needed in association with standard supportive cardiovascular medical therapy, the identification of the specific cause of the disease is crucial. In fact, when an infectious agent is identified, antiviral and/or specific antimicrobial therapy should be started as soon as possible, while in case of autoimmune/immune-mediated forms, immunosuppressive therapy is indicated [7], after ruling out potential contraindications [8].

Although toxic agents account for a minority of causes of myocarditis in Western countries, toxic myocarditis (TM) may display a severe clinical presentation with a heterogeneous prognosis, requiring dedicated diagnostic and therapeutic assessment. To date, detailed reports on histology findings in TM are lacking, especially because of the rarity of biopsy-proven cases. Moreover, TM poses a challenge to clinicians, since robust evidence regarding TM management and prognosis is not available.

Most commonly, TM is due to chemical agents, either used for medical or illicit purposes, or after accidental exposure [9]. Several reports exist regarding TM cases occurring due to established cardiotoxic agents, i.e., cocaine, clozapine, and methamphetamines [10,11,12].

Among cardiotoxic agents, hydrocarbons are rarely reported as TM possible causative agents; still, the relevance of hydrocarbon TM is highlighted by the fact that it is reported to cause more than triple the major adverse outcomes than other cardiotoxic substances (i.e., morphine, hydromorphone, or tramadol) in the United States [13]. Hydrocarbons are organic chemical compounds that contain mainly carbon and hydrogen atoms; they are generally classified as aromatic, characterized by a benzene ring, and aliphatic, which are straight-chained. Moreover, when at least one hydrogen atom is replaced by a halogen, they are defined as halogenated. Hydrocarbons are found in numerous occupational and household products, such as fuels, lubricants, industrial solvents, cleaning agents, paint thinner, and many other products [14]. Hydrocarbons include a variety of compounds, ranging from refrigerant gases to camphor, which can be classified as a terpene.

As with other TM causes, hydrocarbon-mediated myocardial damage may be directly due to hydrocarbon toxicity to the cardiomyocytes and/or mediated by a hypersensitivity reaction, involving adaptive immunity-mediated mechanisms and eosinophilic activation [15]. The release of catecholamines may also play a role in the myocardial damage, as catecholamine-induced TM cases have been reported [16]. Finally, biopsy-proven myocarditis cases caused by the coexistence of viral and toxic agents have not been reported so far.

Thus, since TM clinical features, treatment options, and prognosis have not been elucidated so far, our aims include the following: (1) to describe a case of biopsy-proven myocarditis temporally related to aromatic hydrocarbon exposure, with a focus on the unexpected diagnosis of active viral replication via EMB analysis; (2) to perform a systematic review of the literature regarding clinically suspected or biopsy-proven myocarditis cases related to exposure to hydrocarbon agents.

## 2. Methods

A systematic review of the literature was conducted, aimed at identifying published case reports of myocarditis associated with hydrocarbon exposure. This systematic review followed a pre-specified protocol, according to the preferred reporting items for systematic reviews and meta-analyses (PRISMA) statement (Supplementary Table S1) [17].

Published cases were identified by searching electronic databases (Medline via OvidSP, Medline via PubMed, Medline via Web of Science) from inception to 28 September 2024. Case reports or series of biopsy-proven or clinically suspected hydrocarbon-related myocarditis cases were included. Reports were included if they met each of the following criteria: (1) the type of publication was an original case report, a case series, or a conference abstract, and reviews of the literature were excluded; (2) myocarditis diagnosis, either biopsy-proven or clinically suspected, was based on invasive or non-invasive data after exclusion of alternative causes; (3) exposure to hydrocarbons was considered the causative event.

Exclusion criteria were as follows: (1) myocarditis association with exposure to agents different than hydrocarbons, specifically, benzene derivatives such as drugs, cannabinoids, and endogenous substances; (2) the lack of myocarditis diagnosis in the main text; (3) the lack of a digital object identifier (DOI) for the paper.

Data were extracted from each original report regarding the following: (1) patient demographic characteristics, i.e., sex and age at myocarditis diagnosis, (2) past medical history, (3) chemical characteristic of the hydrocarbon compound (i.e., aromatic, aliphatic, halogenated), (4) cardiovascular symptoms, (5) electrocardiogram (ECG) features at presentation, as well as occurrence and type of arrhythmias during hospital admission, (6) troponin peak during the index hospitalization, (7) echocardiographic findings, (8) coronary angiography, (9) CMR data, if available, (10) EMB data, if available, (11) treatment for myocarditis and/or standard cardiovascular medical therapy, and (12) follow-up data.

Data were extracted in Microsoft Excel (Microsoft Corp, Seattle, WA, USA) through an extraction template. Two authors (T. S. and A. S. G.) extracted the data from each report independently and filled in the extraction form. Disagreements were resolved by discussion; if no agreement was reached, a third author (A.B.) made the decision regarding uncertain cases.

Categorical variables were reported as absolute values and percentages (relative frequencies), and continuous variables were reported using the median and interquartile range (IQR, 1st–3rd quartile). Descriptive statistical analysis was performed using jamovi software (Sydney, Australia, version 2.6.2).

## 3. Case Presentation

**Figure 1:**
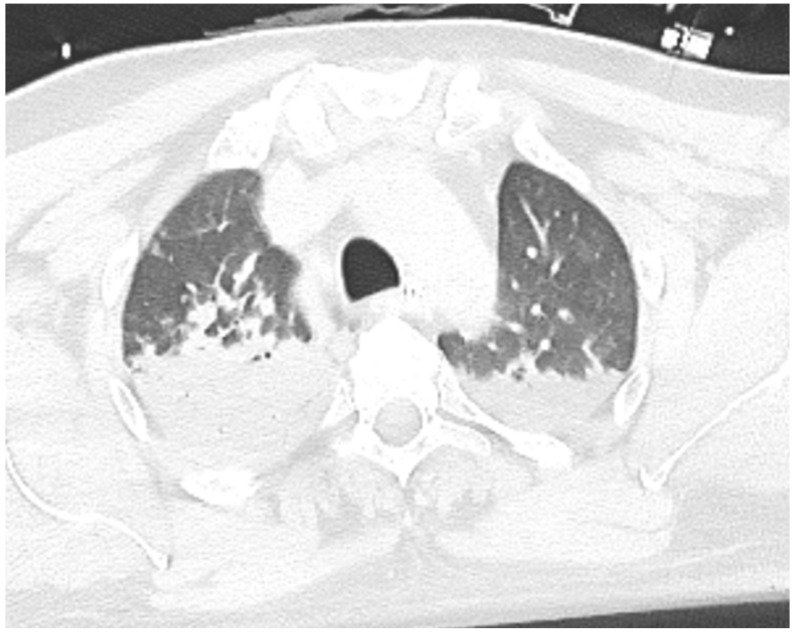
Chest CT scan on admission, showing bilateral diffuse consolidation areas, consistent with chemical pneumonitis.

**Figure 2:**
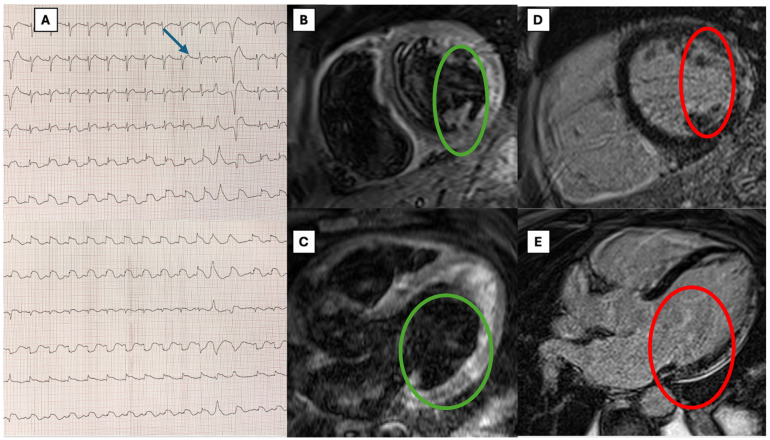
**ECG and CMR findings**. (1) Panel (**A**) shows patients’ ECG on day two after admission: sinus tachycardia is present, together with diffuse ST elevation with “shark fin” morphology, as well as ventricular ectopics (blue arrow). Notably, ECG on day one was unremarkable. (2) Panels (**B**) (short axis) and (**C**) (four chamber) show CMR T2-weighted sequences: subepicardial edema (non-ischemic pattern) is present at the level of the lateral LV wall (green circles). (3) Panels (**D**) (short axis) and (**E**) (four chamber) show CMR T1-weighted post-contrast sequences: subepicardial LGE (non-ischemic pattern) is present at the level of the lateral LV wall, with a corresponding distribution as myocardial edema (red circles).

A 47-year-old male patient, with diabetes mellitus and no other preexisting cardiac or non-cardiac diseases, presented to the emergency department (ED) after falling into a bitumen tank at work. He was diagnosed with chemical pneumonitis, with a high concentration of aromatic hydrocarbons in the urine sample. In the ED, the patient presented severe hypoxemic respiratory failure; the arterial blood gas PaO_2_/FiO_2_ ratio was 100, lactate levels were 7 mmol/L, and carboxyhemoglobin was not detectable. Therefore, he underwent tracheal intubation and was admitted to the intensive care unit. The baseline ECG was unremarkable. A chest CT scan showed bilateral diffuse consolidation areas, consistent with chemical pneumonitis (Figure 1). After pneumological consultation, low-dose IV steroids (methylprednisolone 40 mg IV/day) were therefore administered, with rapid benefit, and the patient was extubated on day two. On the same night, he developed transient hypotension, which required vasoactive therapy with norepinephrine. ECG was repeated, showing sinus rhythm with diffuse ST elevation and ventricular couplets (Figure 2—panel A). Troponin I increased to 1600 ng/L (upper normal limit < 20 ng/L). On urgent echocardiography, severe biventricular disfunction and right ventricular dilatation were noted (left ventricular ejection fraction—LVEF—26%, left ventricular end diastolic volume—LV EDV—58 mL/m^2^, right ventricular fractional area change—RV FAC—16%, tricuspid annular plane systolic excursion—TAPSE—1.56 cm), with mild septal hypertrophy (interventricular septum: 12 mm, LV posterior wall: 9 mm). Intravenous (IV) levosimendan was administered. On day four, the patient developed electrical instability, with frequent non-sustained runs of ventricular tachycardia and one episode of ventricular fibrillation, effectively treated with electrical cardioversion. IV antiarrhythmic therapy with amiodarone and lidocaine at the maximum dosage was started. Progressive hemodynamic compromise with hypotension and initial signs of systemic hypoperfusion (increasing lactate levels, reduced urine output) required the up-titration of inotropic support with dobutamine. Owing to the persistence of refractory sustained ventricular arrhythmias, dobutamine was withdrawn, and on day four, percutaneous stellate ganglion blockade (PSGB) was performed at the patient’s bedside, without complications, in addition to the ongoing antiarrhythmic medication. Urgent coronary angiography showed the absence of coronary artery stenosis.

After two weeks, upon stable improvement of the pneumological condition, a CMR was performed, showing signs of acute myocarditis: on T2-weighted images, myocardial edema was noted at the level of the lateral and inferior LV walls, with a subepicardial non-ischemic pattern; late gadolinium enhancement (LGE) was also present, with a similar distribution; native T1 mapping values and extracellular volume (ECV) were increased (septal T1 1108 ms with normal range 970 ± 70 ms; ECV 34%, normal values < 30%). T2 mapping values were also slightly elevated at the anterolateral medial LV wallT2 62 ms, with normal range 49 ± 5 ms; Figure 2—panels B–E).

**Figure 3:**
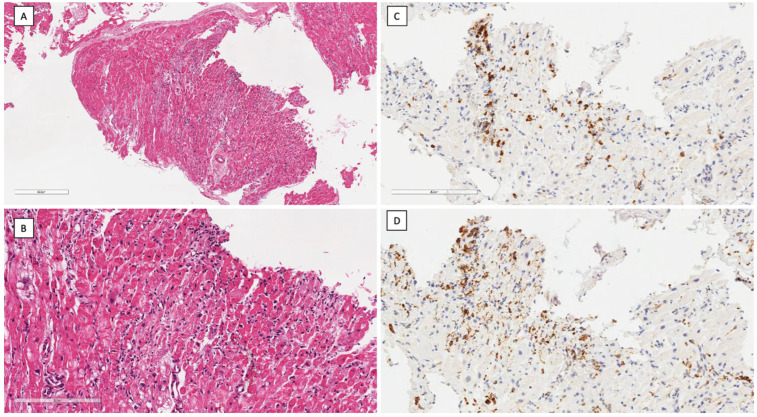
Histology and immunohistochemistry findings from the endomyocardial biopsy: (**A**) massive myocyte necrosis (pale areas) associated with diffuse mononuclear cell infiltrates in the absence of giant cells and eosinophils (hematoxylin–eosin stain, bar = 400 micron); (**B**) higher magnification of B (hematoxylin–eosin stain, bar = 200 micron); (**C**) immunohistochemistry for CD3+ T-cell (>7 cells/mm^2^); (**D**) immunohistochemistry for CD68 macrophages (up to 4 mm^2^; same field as C, bar = 200 micron).

Therefore, on day eighteen, a transfemoral right ventricular EMB was performed, which confirmed acute lymphocytic myocarditis (Figure 3); on molecular analysis, polymerase chain reaction (PCR) unexpectedly revealed parvovirus B19 (B19V) transcriptional activity, with significant viral load (>500 copies/ug). Conversely, PCR analysis on circulating peripheral blood mononuclear cells (PBMC) and plasma revealed B19V, with a viral load <500 copies/ug. Conversely, a PCR search on EMB for adenoviruses, enteroviruses, cytomegalovirus, influenza A/B viruses, Epstein–Barr virus, human herpes virus 6, and herpes simplex was negative.

**Figure 4:**
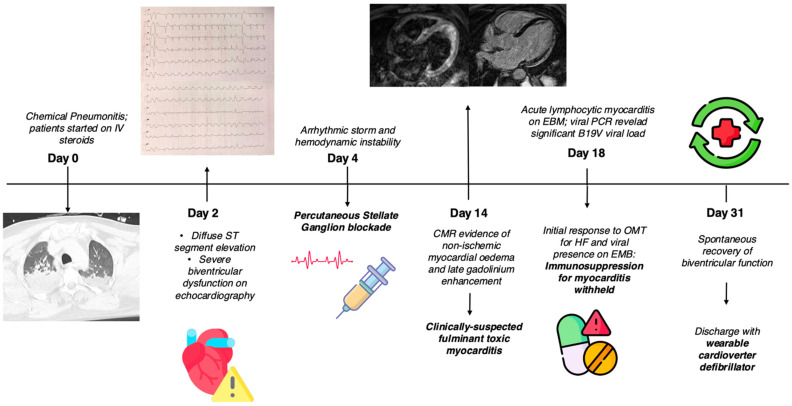
**Timeline of the most relevant clinical and diagnostic findings and therapeutic interventions.** Legend: B19V = parvovirus B19; CMR = cardiac magnetic resonance; EMB = endomyocardial biopsy; HF = heart failure; IV = intravenous: OMT = optimal medical therapy. Created with BioRender Version #201.

On day twenty-eight, echocardiography showed partial improvement of left ventricular function (LVEF 35%) and persistence of severe RV dysfunction and dilatation (FAC 16%, TAPSE 18 mm, RV EDA 16 cm^2^/m^2^). Medical supportive treatment for heart failure with reduced ejection fraction was started, including angiotensin receptor–neprilysin inhibitor, betablocker, mineralocorticoid receptor antagonist, and sodium–glucose transport protein 2 inhibitor. As a result of the microbiological EMB data, consistent with viral myocarditis and the initial recovery of LVEF, immunosuppressive therapy for myocarditis was not started. The patient was discharged on day thirty-one with a wearable cardioverter defibrillator (WCD). At discharge, the ECG was normalized, as well as the C-reactive protein and erythrocyte sedimentation rate. The red blood cell count was within the normal range during the entire hospitalization. Oral low-dose steroid anti-inflammatory therapy was continued for treatment of the pneumonitis (Figure 4).

At the 3-month follow-up appointment in the cardioimmunology outpatient clinic, the patient reported complete resolution of cardiovascular symptoms, was categorized as class I according to the New York Heart Association (NYHA) Functional Classification system, and showed normalization of LVEF (56%), with only mild RV dysfunction (FAC 33%, TAPSE 17 mm). Testing of anti-heart antibodies (AHA) provided negative results [3]. No malignant arrhythmias were detected on 24 h Holter ECG at 3 months, and after multidisciplinary consultation, WCD was therefore discontinued. A pneumological follow-up visit documented a complete remission of the toxic pneumonia; follow-up chest CT at 30 days showed resolution of parenchymal consolidation, and spirometry showed only mild reduction in total lung capacity. A subsequent 6-month follow-up is planned.

## 4. Systematic Literature Review

**Figure 5:**
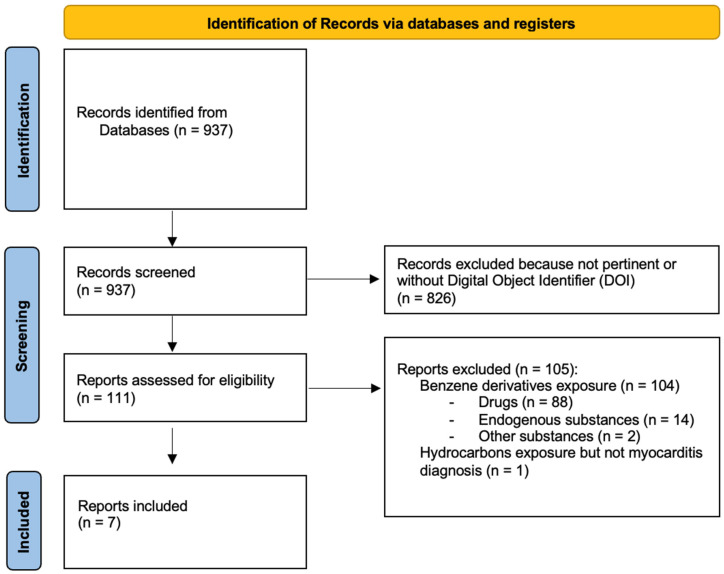
Systematic literature review flowchart.

The flowchart of the systematic literature review selection process of TM cases related to hydrocarbons exposure is reported in Figure 5. The literature search identified 937 references. After the evaluation of each abstract, 111 items were considered eligible for inclusion; thus, these 111 full texts were analyzed in detail. We excluded 104 of these items, 103 because they were related to benzene derivatives (such as drugs, endogenous substances, and cannabinoids alone), and 1 case because myocarditis diagnosis, although clinically plausible, was not explicitly mentioned in the report. Eventually, seven reports were considered for the analysis.

Table 1 illustrates the results of the systematic review of the literature. All patients (7/7) were male and young (median age 24 years, IQR 23–25); only two patients were aged >25 years (i.e., 60 and 77 years old). The causative TM agent was difluoroethane (DFE) in three cases, and a generic halogenated hydrocarbon; a mixture of toluene and aliphatic hydrocarbons; butane; and camphor were the agents in one case each, respectively. Dyspnea (3/7) and chest pain (4/7) were the most frequently reported symptoms at presentation; in one case, dizziness and abdominal discomfort were the symptoms of a paroxysmal complete atrioventricular block (AVB) and unremitting LV systolic dysfunction; in another case, the patient developed cardiogenic shock. ECG at the time of myocarditis diagnosis showed abnormalities in cardiac rhythm (i.e., AVB) and/or ventricular repolarization (i.e., ST segment elevation, T wave inversion, QT prolongation) in 5/7 cases; similarly, troponin was altered in most, but not all, cases (5/7). In three cases, pericarditis was also present and was treated with non-steroidal anti-inflammatory drugs (NSAIDs) in two cases and with colchicine in the remaining one. Moreover, echocardiography showed LV abnormalities in terms of LV dilatation, regional wall motion abnormalities, and/or LVEF reduction in 4/7 cases. With respect to advanced diagnostic techniques, CMR was available in only 2/7 cases, showing myocardial edema in both cases and non-ischemic LGE in one case. In the latter case, CMR was performed twice; after the first episode, when no causative agent had been identified, the patient was readmitted to hospital after 4 months, and CMR was repeated, showing worsening of biventricular function, persistence of edema, and delayed enhancement. EMB was obtained in only one case because of persistent AVB, showing extensive neutrophil and mononuclear cell infiltration, with myocyte necrosis; two weeks after admission, a follow-up EMB was obtained, revealing little residual myocardial inflammation and no residual necrosis. Treatment consisted of general supportive cardiovascular medical therapy in most cases, and in two cases, NSAIDs were used. No patient died, but morphofunctional cardiac abnormalities were still evident at follow-up in one case, possibly indicating incomplete disease resolution. 

## 5. Discussion

The main findings of our systematic review include the following: (1) hydrocarbon-TM is a rare occurrence, but may exhibit a fulminant course and require intensive supportive treatment, (2) prognosis of hydrocarbon-TM may be favorable, provided that an appropriate diagnostic workup is carried out, and (3) EMB is crucial in TM to determine the definitive etiological mechanism and to guide treatment, especially considering its unique role in ruling out the presence of concomitant viral agents in the myocardium.

### 5.1. Peculiarity of Our Case Report

Our case is the first reported biopsy-proven viral myocarditis case diagnosed after a massive exposure to hydrocarbons. To the best of our knowledge, biopsy-proven myocarditis cases in which an association of viral (i.e., confirmed active B19V high viral load on PCR analysis of EMB samples) and toxic causes (i.e., temporal association of ECG changes, troponin elevation, severe ventricular arrhythmia onset, and severe exposure to hydrocarbons) have not been reported so far, either for B19V or other infectious causes, posing challenges to a deeper understanding of etiopathogenetic mechanisms in myocarditis.

The clinical course of our patient was marked by initial respiratory compromise due to chemical pneumonitis, followed by the development of severe cardiac manifestations, including life-threatening arrhythmias and significant myocardial dysfunction. The fact that chemical pneumonia preceded the clinical onset of myocarditis may be related to the peculiar route of exposure to inhaled/aspirated hydrocarbons in this case; when the lung tissue is the first to come into direct contact with the toxic agent, chemical pneumonia may initiate before the agents reaches the cardiovascular system [18]. It has also been reported that hydrocarbons may have disruptive effects on the alveolar surfactant, compromising alveolar function and causing alveolar collapse [19]. Remarkably, it has been hypothesized that some hydrocarbons acquire cardiotoxic properties only after hepatic metabolization, delaying the toxic effect on the heart [20]. This sequence of events explains why pneumonitis is often the first clinical sign observed in hydrocarbon toxicity, followed later by potential cardiac complications. Our case suggests that myocarditis temporally related to hydrocarbon exposure may have an overall good prognosis, similar to that of chemical pneumonia, which may only require supportive treatment and spontaneously resolve after a few weeks.

In our case, the EMB results confirmed the CMR findings of acute myocarditis. Strikingly, the presence of a significant myocardial B19V viral load in the context of acute myocarditis was unexpected but guided the decision against the use of high-dose corticosteroids therapy, reflecting the necessity of thorough diagnostic evaluation before commencing immunosuppressive treatment. The detected viral agent was B19V, which is a vasculotropic virus that can be either virulent (i.e., leading to viral myocarditis) or a non-pathogenic bystander; for suspected B19V-viral myocarditis, the determination of the B19V viral load directly from EMB is essential. In the past, it has been shown that a high B19V viral load, evaluated as B19V>500 copies/ug DNA, was linked to a direct cardiotoxic effect of the virus [21]. Conversely, a recent large retrospective analysis of a prospective patient cohort showed that the determination of B19V transcriptional activity through B19V-RNA evaluation, and not B19V viral load per se, is an unfavorable prognostic predictor in non-ischemic cardiomyopathy patients with B19V-positive EMBs [22].

In this case, even if the determination of B19V active replication through B19V replication intermediates was not possible, we hypothesize that the myocardial damage caused by hydrocarbon toxicity and possibly, the low-dose corticosteroids initiated for pneumonia, may have impaired the native immune surveillance mechanisms and contributed to a clinically significant reactivation of B19V, eventually leading to a severe form of fulminant myocarditis (FM).

EMB is always necessary to determine myocarditis etiology and is even more crucial in FM cases [23]. In fact, non-invasive diagnostic methods such as CMR are not feasible in case of hemodynamic instability and do not allow for a distinction to be made between infectious and non-infectious myocarditis. To date, EMB is the unique diagnostic method than can rule out the presence of viral agents in the myocardium; since EMB is not routinely performed, it is not possible to estimate the prevalence of active viral replication in the context of myocarditis, either non-toxic or toxic, such as in our case.

Thus, in our case, considering both the spontaneous LVEF recovery and EMB findings (i.e., the presence of relevant numbers of B19V copies in the myocardium) led to the decision to withhold immunosuppression. Low-dose IV steroids at anti-inflammatory doses were used and rapidly tapered, since an immune-mediated mechanism was hypothesized, and pneumonitis, indicating the need for steroid administration, was concomitant. Indeed, corticosteroids are used to treat autoimmune myocarditis at high, immunosuppressive doses (i.e., IV methylprednisolone at the dosage of at least 1 mg/kg/day, or equivalent), especially in cases of FM; corticosteroids must therefore be administered for a long period of time [8]. Conversely, lower steroid doses used for shorter times may be useful to treat extracardiac complications in the context of hydrocarbon toxicity, e.g., in form of anti-inflammatory supportive treatment of pneumonitis, such as in this case. Scarce evidence is available regarding the best antiviral treatment for viral myocarditis; in case of active B19 myocardial infection, the use of intravenous immunoglobulin (IVIG) has been tested, with contrasting results [24,25]. New antiviral agents such as telbivudine, a nucleotide antagonist, and interferon are currently being tested [26].

In addition, the use of WCD highlights the importance of a cautious approach when managing the arrhythmic prevention in the post-acute phase of TM. In this high-risk FM myocarditis condition, WCD served as a temporary preventive measure, as well as allowed for continuous arrhythmia monitoring until the patient’s cardiac function was restored.

In addition, our case is also the first known case of fulminant myocarditis temporally associated with hydrocarbon exposure, requiring hemodynamic support with inotropes and vasopressors, and intensive arrhythmic treatment including PGSB. At onset, biventricular function was severely impaired, but after 3 months, LVEF completely normalized, and RV function significantly improved. This case highlights the critical role of PGSB in managing refractory catecholamine-sensitive arrhythmias in the setting of FM [27], especially when used at the initial stages of the disease [28]. Another important factor is that inotropic support with dobutamine had to be stopped, since its pro-arrhythmic effects could have contributed to the exacerbation of ventricular arrhythmias.

Finally, our case highlights the importance of multidisciplinary management in cardioimmunology. This is key not only for the management of myocarditis (i.e., a multidisciplinary team involving cardiologists, immunologists, pathologists, and infectious diseases specialists) but also regarding the entire spectrum of hydrocarbon systemic toxicity (i.e., pneumologists).

### 5.2. Evidence from the Systematic Literature Review

Aromatic hydrocarbons are recognized for their cardiotoxic effects, which include disruption of myocardial ion channels and increased sensitivity to catecholamines, predisposing individuals to arrhythmias and exerting negative inotropic effects [29]. In the literature, the cardiotoxic effects of hydrocarbons, especially those present in petroleum products and industrial solvents, are apparently worse than those of other chemical agents [30], even if it is difficult to elucidate whether a sizable proportion of the reported major adverse cardiovascular events and even deaths are a direct consequence of the cardiotoxic, rather than the result of systemic multiorgan toxicity.

The systematic review showed that very few cases of TM associated with hydrocarbons have been reported so far, with those reported mainly involving halogenated hydrocarbons that are often found in household products. TM patients were exclusively young men; this could be related to the type of exposure (inhalant substance abuse) but also to the reported increased incidence of clinically suspected myocarditis with infarct-like presentation among young males [2]. Various mechanisms have been hypothesized for this epidemiological trend, including the role of sex hormones, with estrogens exerting a blunting effect on the expression of various inflammatory cell in animal models of infectious myocarditis [31].

Among the collected reports, the prognosis for hydrocarbon-TM was mostly good, with the restoration of biventricular systolic function and/or the resolution of arrhythmias at short-term follow-up observed in most cases, and no deaths were reported. This may be linked to the fact that the reported treatment was heterogeneous; in fact, while robust evidence supports the use of immunosuppression in fulminant [23] and non-fulminant virus-negative EMB-proven myocarditis [8,32], the role of antiviral agents is still debated for virus-positive cases [26], and specific recommendations for EMB-proven TM cases are lacking. The type and duration of treatment of TM is currently based on a case-by-case approach, with relevant heterogeneity among different centers in terms of drugs choices and tapering duration. The cornerstone of TM treatment lies in eliminating exposure to toxic agents, whether resulting from accidental contact or therapeutic drug use. With respect to medical supportive treatment, the use of NSAIDs is not endorsed in myocarditis in general, excluding cases with pericardial involvement [33]; indeed, in animal studies, a variety of cardiac toxic effects from NSAIDs have been reported, including exacerbation of inflammatory infiltrates [34] and long-term adverse cardiac remodeling [35]. For cases with hemodynamic and/or arrhythmic instability, supportive treatment with inotropes, anti-arrhythmic drugs, and eventually, mechanical circulation support in the setting of the cardiac intensive care unit is necessary.

In some of the cases from the literature review, troponin and/or ECG were normal; this is in agreement with previous findings in myocarditis patients, since no single non-invasive examination is capable of supporting the diagnosis of myocarditis, and a normal ECG or troponin dosage cannot rule out myocarditis [2].

The main symptoms were dyspnea and chest pain, but a single case of hydrocarbon-TM with arrhythmic presentation in the form of both hypo- and hyperkinetic ventricular arrhythmias has been reported. In that case, due to the presence of complete atrioventricular block, the use of a temporary pacemaker was necessary; after 3 days, restoration of normal atrioventricular conduction led to the possibility of pacemaker removal. Also in that case, a control EMB confirmed the reduction in the inflammatory infiltrates in the myocardium, alongside the improvement of the patient’s clinical conditions. While complete heart block is an uncommon presentation of myocarditis, giant cell myocarditis and cardiac sarcoidosis are more frequently associated with such a presentation, requiring aggressive immunosuppression and surveillance for major cardiac complications; however, several cases of lymphocytic virus-negative and virus-positive myocarditis with complete heart block at presentation have been reported [1].

CMR may underdiagnose myocarditis [36], especially in cases with heart failure or arrhythmic presentation, probably due to edema re-absorption and prevailing myocardial apoptosis rather than necrosis. In addition, CMR sensitivity/specificity has not been specifically tested in the setting of TM, which is also infrequently used as animal model of myocarditis, with murine-induced viral and/or autoimmune myocarditis being the most common experimental models [37].

## 6. Study Limitations

In our study, a selection bias towards the inclusion of severe myocarditis cases may be present, with possible underestimation of unpublished less severe TM cases. Secondly, within the case reports included in our systematic review, follow-up was heterogeneous in terms of time, and long-term follow-up was not available for any of the included cases, leading to a possibly inaccurate description of long-term prognostic data. Thirdly, detection of B19V RNA intermediates such as mRNAs, which have recently been identified as potential diagnostic markers to differentiate latent B19V infection and significant transcriptionally active B19V-infection of the myocardium [4,38], was not possible in our case. PCR data on the B19V at follow-up were not available because a follow-up EMB was not clinically and guideline indicated due to the concomitant recovery of biventricular function and the absence of arrhythmia, although this information might have been interesting for better understanding the disease pathophysiology.

Finally, the range of years of publication of the included reports was wide, possibly resulting in a heterogeneity in the available diagnostic modalities and types of chemical compounds causing TM.

## 7. Conclusions

We report the first case of a fulminant viral myocarditis temporally associated with aromatic hydrocarbons exposure due to a coexistence of two pathogenic mechanisms (i.e., viral and toxic). Our case report and systematic literature review emphasize the crucial role of a comprehensive diagnostic evaluation, primarily including prompt EMB execution and its histological and microbiological evaluation, specifically for the identification of the presence of a concomitant high myocardial viral load, in driving therapeutic choices. Our study highlights the rarity of hydrocarbon-induced TM, which may exhibit a fulminant course and require intensive cardiological treatments, including advanced management of both hypokinetic and hyperkinetic arrhythmias. Our case and the data derived from this systematic literature review also show that hydrocarbon-TM prognosis may be good, with potential for recovery if a guideline-based diagnostic and therapeutic approach is followed. Given the limited long-term follow-up data, further studies are needed to characterize hydrocarbon-TM prognosis and describe the standardized treatment protocols.

## Figures and Tables

**Table 1:** **Systematic literature review results and data for the index case.** Cases of hydrocarbon-related TM were systematically searched among multiple databases, and main clinical and imaging findings were collected according to a pre-specified protocol. Data of our case report have also been included (labeled as “index case”). AF = atrial fibrillation; ARNI = angiotensin receptor/neprilysin inhibitor; CAG = coronary angiogram; CMR = cardiac magnetic resonance; DM = Diabetes Mellitus; EBM = endomyocardial biopsy; FU = follow-up; iSGLT2 = SGLT2 inhibitors; LGE = late gadolinium enhancement; LV EF = left ventricle ejection fraction; M = male; MRA = mineralocorticoid receptor antagonist; N.A. = not available; NSVTs = non sustained ventricular tachycardia; PSGB = Percutaneous Stellate Ganglion blockade; PVB19 = parvovirus B19; PVCs = premature ventricular contractions; RBBB = right bundle branch block; RV EDA = right ventricle end diastolic area; RV FAC = right ventricle fractional area change; TWI = T waves inversion; VF = ventricular fibrillation. ^1^ Main exposure route was inhalation; ^2^ Exposure route was inhalation and skin contact; ^3^ Final diagnosis was myopericarditis; ^4^ Exposure route was ingestion.

Authors	Age (Years)	Sex	Past Medical History	Causative Agents ^1^	Symptoms and Signs	ECG and Rhythm Monitoring	TnI Peak	Echocardiography	CAG	CMR	EBM	Therapy	FU	Death
Rodrigues et al., 2019	77	M	AF	Generic halogenated hydrocarbon	Palpitations, chest pain, dyspnoea	N.A.	Neg	LV EF 33% with diffuse hypokinesia	Neg	N.A.	N.A.	Supportive therapy	After 5 months: LVEF 43%, NHYA I	no
Jolly et al., 2021	25	M	Neg	Difluoroethane (DFE) + marijuana	Chest pain, nausea and vomiting	Inferolateral ST elevation	2.06 ng/mL	LV EF 40–45%	Neg	Edema in the mid and apical LV, no LGE	N.A.	Colchicine ^3^	After 6 weeks: LVEF 60%	no
Dinsfriend et al., 2016	23	M	Substance abuse, bipolar disorder	Difluoroethane (DFE)	Palpitation, pericarditic chest pain, dyspnoea and diaphoresis	Incomplete RBBB	0.13 ng/mL	N.A.	N.A.	First CMR: biventricular dilation with preserved EF, subepicardial and midwall LGE, edema.	N.A.	High-dose ibuprofen ^3^	Relapse at 4 months; second CMR: RV EF 47%, LV EF 51%, with persistent edema and LGE	no
Dingle et al., 2018	24	M	N.A.	Difluoroethane (DFE)	Dyspnoea and imbalance	Diffuse ST segment elevation and PR depression; frequent PVCs	1.89 ng/mL	Normal	N.A.	N.A.	N.A.	Beta-blocker	N.A.	no
Bayar et al., 2013	20	M	N.A.	Butane gas	Chest pain related to different positions	Diffuse ST segment elevation	3.5 ng/mL	Normal	Neg	N.A.	N.A.	Indomethacin ^3^	N.A.	no
A. T. Knight et al., 1991	20	M	Upper respiratory infection in the last four months	Mixture of toluene and aliphatic hydrocarbons ^2^	Nausea, vomiting, lethargy, right-sided abdominal pain	TWI, intermittent 2:1 AVB; 4 s of asystole; VT and VF	N.A.	LVEF 35% with dyssynergy of the distal part of the septum	N.A.	N.A.	First EBM: extensive neutrophil and mononuclear cell infiltration and myocyte necrosis; second EBM: little residual inflammation and no necrosis	Temporary pacemaker	Three days after admission, control ECG showed a normal pattern and the pacemaker was removed	no
M. Bhaya et al., 2007	60	M	Anemia	Camphor ^4^	Nausea, vomiting; cardiogenic shock at presentation	RBBB, QT prolongation	28.1 ng/mL	Biventricular dilatation, LVEF 40% with diffuse hypokinesia, right ventricle disfunction	N.A.	N.A.	N.A.	Norepinephrine for 12 h	Within 12 h, hemodynamics improved, and ECG normalized Echo after two-weeks: normal biventricular dimensions and function	no
Index case	47	M	DM	Aromatic hydrocarbons	Dyspnea	Diffuse ST elevation, NSVTs, and one episode of VF	1.6 ng/mL	Severe biventricular dysfunction and right ventricular dilatation (LV EF 26%, RV FAC 16%, RV EDA 16 cm^2^/m^2^)	Neg	Edema and subepicardial LGE on lateral and inferior wall of LV; T1 mapping and extracellular volume increased	Acute lymphocytic myocarditis; molecular analysis revealed positive B19V transcriptional activity with significant viral load (>500 copies/μg)	Antiarrhythmic therapy with amiodarone and lidocaine; PSGB, vasoactive therapy with norepinephrine; inotropic support with levosimendan and dobutamine; heart failure treatment: ARNI, betablocker, MRA, and iSGLT2	At 3-month follow-up: class I NHYA; echo with normalization of LVEF; no malignant arrhythmias at 24 h Holter ECG	no

## Data Availability

Not applicable.

## Supplementary Materials

The following supporting information can be downloaded at: https://www.mdpi.com/article/10.3390/ijms26094006/s1. Reference [39] is cited in Supplementary Materials.

## Abbreviations and Acronyms

AHA	anti-heart antibodies
CMR	cardiac magnetic resonance
EMB	endomyocardial biopsy
PCR	polymerase chain reaction
TM	toxic myocarditis
